# Reassessment of Mendelian gene pathogenicity using 7,855 cardiomyopathy cases and 60,706 reference samples

**DOI:** 10.1038/gim.2016.90

**Published:** 2016-08-17

**Authors:** Roddy Walsh, Kate L. Thomson, James S. Ware, Birgit H. Funke, Jessica Woodley, Karen J. McGuire, Francesco Mazzarotto, Edward Blair, Anneke Seller, Jenny C. Taylor, Eric V. Minikel, Exome Aggregation Consortium, Daniel G. MacArthur, Martin Farrall, Stuart A. Cook, Hugh Watkins

**Affiliations:** 1 NIHR Royal Brompton Cardiovascular Biomedical Research Unit, Royal Brompton Hospital and Imperial College London, London, UK; 2 National Heart and Lung Institute, Imperial College London, UK; 3 Oxford Medical Genetics Laboratory, Oxford University Hospitals NHS Foundation Trust, The Churchill Hospital, Oxford, UK; 4 Radcliffe Department of Medicine, University of Oxford, Oxford, UK; 5 MRC Clinical Sciences Centre, Imperial College London, UK; 6 Laboratory for Molecular Medicine, Partners HealthCare Personalized Medicine, Cambridge, Massachusetts, USA; 7 Department of Pathology, Massachusetts General Hospital and Harvard Medical School, Boston, Massachusetts, USA; 8 Department of Clinical Genetics, Oxford University Hospitals NHS Foundation Trust, The Churchill Hospital, Oxford, UK; 9 Oxford NIHR Biomedical Research Centre, Oxford, UK; 10 The Wellcome Trust Centre for Human Genetics, University of Oxford, Oxford, UK; 11 Analytic and Translational Genetics Unit, Massachusetts General Hospital, Boston, Massachusetts, USA; 12 Broad Institute of MIT and Harvard, Cambridge, Massachusetts, USA; 13 Program in Biological and Biomedical Sciences, Harvard Medical School, Boston, Massachusetts, USA; 14 Exome Aggregation Consortium (ExAC), Cambridge, Massachusetts, USA; 15 Department of Medicine, Harvard Medical School, Boston, Massachusetts, USA; 16 National Heart Centre Singapore, Singapore; 17 Duke–National University of Singapore, Singapore

**Keywords:** clinical genetics, Exome Aggregation Consortium, inherited cardiomyopathy, Mendelian genetics, variation interpretation

## Abstract

**Purpose:**

The accurate interpretation of variation in Mendelian disease genes has lagged behind data generation as sequencing has become increasingly accessible. Ongoing large sequencing efforts present huge interpretive challenges, but they also provide an invaluable opportunity to characterize the spectrum and importance of rare variation.

**Methods:**

We analyzed sequence data from 7,855 clinical cardiomyopathy cases and 60,706 Exome Aggregation Consortium (ExAC) reference samples to obtain a better understanding of genetic variation in a representative autosomal dominant disorder.

**Results:**

We found that in some genes previously reported as important causes of a given cardiomyopathy, rare variation is not clinically informative because there is an unacceptably high likelihood of false-positive interpretation. By contrast, in other genes, we find that diagnostic laboratories may be overly conservative when assessing variant pathogenicity.

**Conclusions:**

We outline improved analytical approaches that evaluate which genes and variant classes are interpretable and propose that these will increase the clinical utility of testing across a range of Mendelian diseases.

*Genet Med* **19** 2, 192–203.

## Introduction

Interpretation of rare genetic variation, whether in a clinical diagnostic or research setting, has not kept pace with the accelerating data generation using high-throughput DNA sequencing. Increasingly extensive gene panels, as well as whole-exome and genome sequencing, are used to interrogate the growing number of genes implicated in Mendelian diseases.^1^ However, such panels only modestly increase the number of high-confidence diagnostic results while identifying ever larger numbers of variants of uncertain significance^2,3^; these inconclusive results not only reduce the clinical utility of testing but also can lead to misinterpretation and misdiagnosis.

Central to the challenge of rare variant interpretation is the paradox that individually rare variants are now seen to be collectively common. Although it is accepted that a common variant can be excluded as a cause of a rare and penetrant Mendelian disease, the community has been slower to recognize that many rare variants identified in Mendelian disease genes are innocent bystanders and some “rare” variants are not rare at all. Recent population sequencing efforts have raised awareness of these issues (e.g., the 1000 Genomes Project,^4^ the Exome Sequencing Project (http://evs.gs.washington.edu/EVS), but the full extent is now revealed in the Exome Aggregation Consortium (ExAC) data set (http://exac.broadinstitute.org), in which the average exome contains 7.6 rare nonsynonymous variants (minor allele frequency (MAF) <0.1%) in well-characterized dominant disease genes, with the majority being very rare or “private.”^5^ Clearly, only a small minority can actually cause a penetrant Mendelian disease.^6^

The challenges of variant interpretation in Mendelian disorders are particularly well illustrated by inherited cardiomyopathies: hypertrophic cardiomyopathy (HCM), dilated cardiomyopathy (DCM), and arrhythmogenic right ventricular cardiomyopathy (ARVC). These largely autosomal dominant disorders are relatively common, genetically heterogeneous, and medically important;^7^ consequently, cardiomyopathy genes feature prominently in the American College of Medical Genetics and Genomics list of proposed genes to be routinely analyzed in all exome or genome sequencing.^8^ Although clinical genetic testing in cardiomyopathy has been available for more than a decade, the number of genes reported as disease-causing has increased dramatically in recent years, often without robust evidence.

Here, we leveraged two substantial resources to better understand and interpret rare variation in cardiomyopathy genes. We compared sequence data from 7,855 individuals who had a clinical diagnosis of cardiomyopathy with 60,706 reference samples from the ExAC consortium, the first data set powered to assess variant alleles present in the population at a range of 1:1,000–100,000 that might previously have been considered pathogenic yet may in fact be too common to cause penetrant Mendelian disease.

Through these analyses, we aimed to define the genes, regions of genes, and/or classes of variants that can be reliably interpreted in a clinical setting and in doing so enhance variant interpretation and increase clinical diagnostic yields.

## Materials and Methods

### Clinical and control cohorts

Data from 3,267 individuals with a clinical diagnosis of HCM, 559 with DCM, and 361 with ARVC were obtained from the Oxford Medical Genetics Laboratory for up to 16 genes for HCM, 28 genes for DCM, and 8 genes for ARVC (**Supplementary Table S1a** online). Data from Partners Laboratory of Molecular Medicine (LMM) were downloaded from the supplemental files of previous publications and included data from up to 18 genes sequenced in 632–2,912 HCM patients^9^ and up to 46 genes sequenced in 121–756 DCM patients^3^ (**Supplementary Table S1b** online). Because there were no significant differences in the proportion of cases of rare variants in each gene between the two laboratories (Fisher's exact test), the data were combined (**Supplementary Table S3a,b** online).

Data were downloaded from ExAC (http://exac.broadinstitute.org; version 0.3, January 2015). Only genes with a high proportion of coding regions covered to a median sequence depth of >30× and only high-quality (PASS filter) variants were included in our analyses. In addition, we adjusted the total number of ExAC samples per gene based on the mean coverage at the variant sites of interest.

Please refer to **Supplementary Note S1** online for more detailed information on each component cohort.

### Defining an allele frequency threshold for rare variation

The single most common confirmed pathogenic variant in both clinical cohorts was *MYBPC3* c.1504C>T (p.Arg502Trp), which was found in 104/6,179 HCM cases (1.7%; 95 CI: 1.4–2.0%); this variant was only observed three times in ExAC (MAF 2.5 × 10^−5^). We therefore applied a MAF threshold of 1 × 10^−4^ as a conservative upper bound because variants more frequent than this in the general population would not be expected to be highly penetrant pathogenic mutations (see **Supplementary Note S2** online). This MAF does not exclude the possibility of more common deleterious founder variants in specific populations where the genetic architecture of cardiomyopathy is not well defined.

### Calculation of rare variant frequency in cardiomyopathy and ExAC cohorts

For each gene, the frequency of rare variants (MAF <1 × 10^−4^) in ExAC was calculated by dividing the sum of the adjusted allele count by the mean of the total adjusted alleles. The frequency of rare variation in the cardiomyopathy cohorts was calculated by dividing the sum of rare variants identified in cardiomyopathy cases by the total number of patients analyzed for each gene. Only likely protein-altering variants in designated canonical transcripts (**Supplementary Table S2** online) were analyzed: missense, in-frame insertions/deletions, frameshift, nonsense, and variants affecting the splice donor and acceptor regions (first and last two bases of each intron). Analyses were performed for all protein-altering variants and separately for variants predicted to be nontruncating (missense and in-frame insertions and deletions) and truncating (frameshift, nonsense, splice donor/acceptor). To ensure that population-specific variants did not have a confounding effect on this analysis, we compared the results seen in the LMM DCM cohort for all samples and for Caucasians only (see **Supplementary Note S4** online).

In total, after data from both clinical laboratories were combined and poorly covered genes in ExAC were excluded, 20 genes sequenced in 632–6,179 HCM patients, 46 genes sequenced in 121–1,315 DCM patients, and 8 genes sequenced in 93–361 ARVC patients were analyzed. See **Supplementary Table S2** online for full details of cohort sizes for each gene.

### Comparison of variation between cardiomyopathy and ExAC cohorts

For each gene, the frequency of rare variation in the clinical cohort was compared with that in ExAC. Case excess was defined by subtracting the proportion of individuals in ExAC with a filtered variant from the proportion in the clinical cohort. We made the simplifying assumption that the frequency of rare benign variants was equivalent in cases and ExAC, and that the frequency of pathogenic variants in ExAC is sufficiently low so as not to affect this comparison. A Fisher's exact test was performed to test the significance of observed excess in cases.

For each gene and variant class, we calculated two related metrics: the odds ratio (OR) with 95% confidence intervals and the etiological fraction (EF)^10,11,12^ (calculated as: (OR-1)/OR × 100; for further information on EF please refer to **Supplementary Note S3** online). These metrics were calculated for all protein-altering variants and separately for predicted nontruncating and truncating subsets.

All statistical tests used in these analyses are two-sided, unless otherwise stated, and analysis was undertaken using Stata statistical analysis software (StataCorp. 2007. Stata Statistical Software: Release 10. College Station, TX: StataCorp LP.).

### Distribution of missense variants in MYH7

To identify putative hotspots of pathogenic missense mutations in *MYH7*, distinct rare missense variants from the HCM, DCM, and ExAC cohorts were mapped along the protein sequence. Nonrandom mutation cluster (NMC),^13^ implemented in the iPAC Bioconductor R package, was used to identify clusters of variants in each cohort (R source code of NMC algorithm: https://bioconductor.org/packages/release/bioc/html/iPAC.html).

### Analysis of research cardiomyopathy cohorts

Research cardiomyopathy cohorts are defined as published studies from research laboratories where patient samples were subjected to sequencing across panels of cardiac genes. The HCM research cohort^14^ sequenced 874 patients across 35 genes (12 associated primarily with HCM, 7 with DCM, 7 with ARVC, and 9 with arrhythmias, as stated by Lopes et al.). The DCM research cohort^15,16,17^ comprised 312–324 patients sequenced for 12 confirmed and putative DCM genes (not including TTN). Putative pathogenic variants in these studies are not identified by clinical-grade classification but rather by criteria such as variant type, population frequency, and in silico algorithm prediction—the details of the criteria used in each study are described in the published articles.

Rare variant frequencies and case excess were calculated for each gene as described for the clinical cohorts. The number of variants reported to be putatively pathogenic for each gene in these studies was compared with the number predicted to be pathogenic based on the case excess observed in these cohorts.

### HGMD cardiomyopathy mutations in ExAC

Variants in the Human Genome Mutation Database (HGMD; professional version 2015.1) associated with HCM, DCM, or ARVC (“disease-causing mutations” with a HGMD tag of DM and DM?) were identified based on manual curation of the HGMD disease terms. The total allele frequency and count from ExAC were extracted for each variant. Polymorphisms (ExAC MAF >1 × 10^−2^) were removed from the analysis. The number of HGMD variants present in ExAC was calculated at any frequency and with MAF >1 × 10^−4^. The total number of ExAC alleles and the total number of ExAC individuals with HGMD-associated cardiomyopathy variants were also calculated for each disease. Additionally, the ExAC frequencies of HGMD cardiomyopathy variants previously observed only once in the Exome Sequencing Project were analyzed to assess how the enhanced resolution of ExAC can clarify previously uninterpretable variants.

## Results

### Comparison of rare variation between cardiomyopathy cohorts and ExAC

Variants identified by sequencing of putative cardiomyopathy genes in cases (*n* = 7,855) were collated by disease and gene (**Supplementary Table S1a,b** online). We compared the burden of rare protein-altering variants (ExAC MAF <1 × 10^−4^) detected in 20 HCM genes, 48 DCM genes, and 8 ARVC genes in HCM, DCM, and ARVC cases, respectively, with the burden observed in ExAC. Predicted truncating and nontruncating variants were analyzed separately (**Table 1** and **Supplementary Table S4a–c** online).

**Figure 1:**
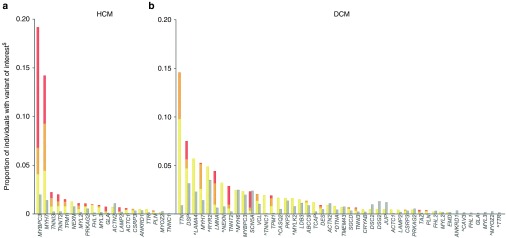
**Proportion of individuals with rare variants in hypertrophic cardiomyopathy (HCM) and dilated cardiomyopathy (DCM) in combined clinical cohorts (data from Oxford Medical Genetics Laboratory and Laboratory of Molecular Medicine) compared with Exome Aggregation Consortium (ExAC) data (gray columns).** Variants counted were single-nucleotide changes or small insertion/deletion variants detected in the coding region ±2 bp, with an ExAC minor allele frequency of <1 × 10^−4^ (see Methods). Clinical cohorts: HCM, *n* = 632 to 6,179 and DCM, *n* = 121 to 1,315 (see Table 1, **Supplementary Table S5a,b** online). Information on reported pathogenicity class (red = pathogenic (P), orange = likely pathogenic (LP), yellow = variant of uncertain significance (VUS)) is overlaid. See **Supplementary Tables S1, S4a,b** online for full details. ^ = genes analyzed in fewer than 200 cases. ExAC: *n* = mean of total adjusted allele count for rare variant carriers. For HCM genes, *n* ranged from 47,153 to 60,647; for DCM genes, *n* was 42,697 to 60,647 (see **Supplementary Table S5a,b** online). CTF1 and RBM20 were removed from analysis due to poor coverage in ExAC.

As expected,^18,19,20^ rare variation in the two major HCM genes accounted for the majority of variation in HCM cases (*MYBPC3*, 19.0% of cases; *MYH7*, 14.2%). Rare variants were less numerous in other well-characterized HCM genes (*TNNI3*, *TNNT2*, *TPM1*, *MYL2*, *MYL3*, *ACTC1*, *PLN*) and phenocopy genes (*GLA*, *LAMP2*, *PRKAG2*) (≤2% cases per gene). For each of these genes there is a significant (*P* < 0.05 after Bonferroni correction) excess of variation in cases as compared with ExAC, thus confirming their association with disease (**Figure 1** and **Table 1** and **Supplementary Table S5a** online). However, for several more recently reported HCM genes (*TNNC1*^21^, *MYOZ2*^22^, *ACTN2*^23^, *ANKRD1*^24^) there was no significant excess of rare genetic variation in these HCM cases.

DCM is highly genetically heterogeneous, with up to ~60 implicated genes.^20,25,26^ In the clinical cohorts, truncating variants in *TTN* were most common (14.6%), in accordance with our findings in large research cohorts.^27,28^ The prevalence of rare variants in other well-characterized DCM genes was modest (*MYH7*, 5.3%; *LMNA*, 4.4%; *TNNT2*, 2.9%; and *TPM1*, 1.9%) but significantly enriched compared with ExAC (**Figure 1** and **Table 1** and **Supplementary Table S5b** online). However, with the exception of truncating variants in *DSP* (2.8%), there was limited burden and modest or no significant excess variation in the remaining 40 genes tested. In ARVC, the five major genes each showed significant excess in cases (**Table 1** and **Supplementary Table S5c** online).

Overall, the yield of pathogenic (P) and likely pathogenic (LP) variants was 32% for HCM, 13% for DCM (but note that *TTN* was only sequenced in one-third of samples), and 36% for ARVC. Of note, in the genes robustly supported by an excess of pathogenic and likely pathogenic variants, even variants of uncertain significance were seen in excess over ExAC, suggesting that clinical laboratories may be overly conservative (**Figure 1**).

### Interpretation of variation by gene and variant class

Many variants in confirmed disease genes can be interpreted with confidence based on cumulative experience (e.g., multiple occurrences of segregation in families, de novo mutations, founder variants) and/or functional insights (e.g., null alleles in haploinsufficient genes). However, our ability to evaluate the pathogenicity of novel variants depends on the signal-to-noise ratio. For each gene and variant class, we calculated two related metrics: the odds ratio (OR) (ratio of odds of cardiomyopathy comparing rare variant carriers with noncarriers) and the etiological fraction (EF), which is a commonly used measure in epidemiology^10,11,12^ that estimates the proportion of cases in which the exposure (in this case, a rare variant in a gene) was causal (see **Supplementary Note S3** online).

**Figure 2:**
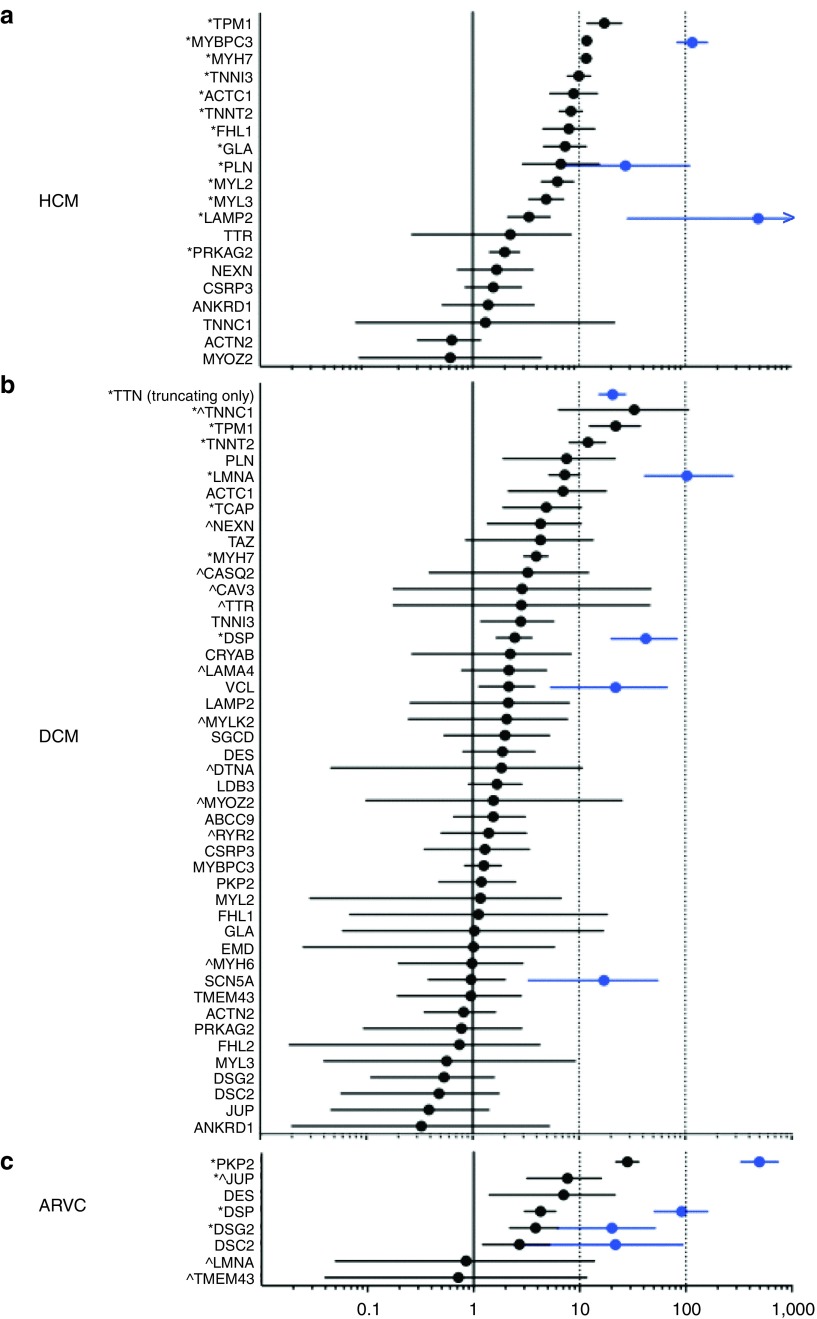
**Odds ratios (OR) with 95% confidence interval (CI)** for each gene tested in the hypertrophic cardiomyopathy (HCM) (*n *= 632 to 6,179), dilated cardiomyopathy (DCM) (*n *= 121 to 131), and arrhythmogenic right ventricular cardiomyopathy (ARVC) (*n*= 93 to 361) clinical cohorts compared with Exome Aggregation Consortium (ExAC) reference samples (*n* = mean of total adjusted allele count for rare variant carriers. for HCM genes, *n* = 47,153 to 60,647; for DCM genes, *n* = 42,697 to 60,647; and for ARVC genes, *n* = 51,126 to 60,218). See **Supplementary Table S5a–c** online for data used to generate this plot. Data have been plotted (log_10_ scale) for all protein-altering variants (black) and truncating variants (blue). For truncating variants, OR with 95% CI have been plotted for genes where a statistically significant difference was observed for this variant type on FET. *Statistically significant Fisher's exact test (FET) (*P*=0.05 with Bonferroni correction; HCM *P* ≤ 0.0025; DCM *P* ≤ 0.001; and ARVC *P* ≤ 0.006.). ^Genes analyzed in fewer than 200 cases. CTF1 and RBM20 were removed from analysis owing to poor coverage in ExAC.

These analyses reaffirm high ORs and EFs in key cardiomyopathy genes and also highlight a number of previously reported cardiomyopathy genes that show limited disease association when compared with a very large number of reference samples (**Figure 2** and **Table 1** and **Supplementary Table S5a–c** online). As expected, many genes have divergent results for truncating as compared with nontruncating variants; for example, *MYH7* has an OR of 1 (0.5–4.5) for truncating variants versus an OR of 12 (10.9–13.3) for nontruncating variants in HCM cases.

This observation confirms the widely accepted view that missense alleles of *MYH7* act as dominant negatives in HCM whereas truncating variants are not pathogenic. In genes whose truncating alleles are disease-causing, ORs are typically higher owing to the lower rate of truncating variants in the population. As expected, truncating variation in *MYBPC3* associates strongly with HCM (the result of haploinsufficiency^29^), but neither truncating nor nontruncating variants in *MYBPC3* show a significant association with DCM (OR = 1.3 (0.8–1.8); EF = 0.21 (0–0.46)), which is a finding that fits with mechanistic insights but challenges some widely held viewpoints.^15,30^ Among the ARVC genes, truncating alleles are informative for four major genes (and particularly common for *PKP2* and *DSP*), whereas nontruncating variants in these genes are difficult to interpret reliably (**Figure 2** and **Table 1** and **Supplementary Tables S4c and S5c** online).

### Using protein domain knowledge to improve variant interpretation

**Figure 3:**
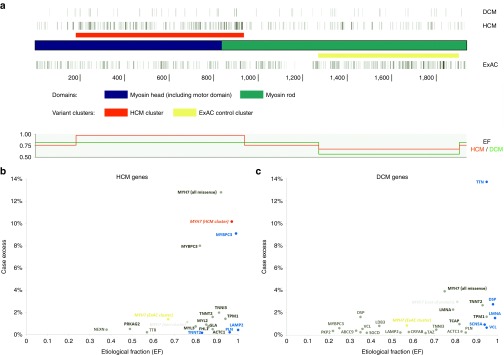
**Variant interpretation.** (**a**) Distribution of rare (Exome Aggregation Consortium (ExAC) minor allele frequency <1 × 10^−4^) MYH7 missense variants in hypertrophic cardiomyopathy (HCM) (*n *= 864) and dilated cardiomyopathy (DCM) (*n *= 69) clinical cohorts and ExAC (*n* = 816), with the key myosin protein regions highlighted. Using nonrandom mutation cluster analysis (NMC), (refer to **Supplementary Methods** online), a significant variant cluster (*P* < 3 × 10^−15^, false discovery rate (FDR), q < 5 × 10^−^13) was identified between residues 181 and 937 (involving the motor domain, lever arm, and part of the rod) in HCM cases, and depletion in this region and a significant cluster (*P* < 3 × 10^−8^, FDR q < 4 × 10^−5^) was identified between residues 1,271 and 1,903 (in the part of the rod that forms the filament backbone) in ExAC samples. The etiological fraction (EF) for a rare MYH7 missense variant identified in a HCM proband ranges from 0.97 in the HCM cluster to 0.67 in the control cluster. Vertical gray bars depict the positions of variants in cohorts; grayscale shows variant density where variants are coincident. An overview of the genetic landscape of HCM (**b**) and DCM (**c**) for truncating (blue) and nontruncating (gray) variants, as well as *MYH7* missense variants in the clusters identified in (**a**) (orange, disease cluster; yellow, ExAC control cluster). The case excess (y-axis) is the frequency of rare variation in disease cohorts over and above the frequency in ExAC and indicates the relative importance of the gene and variant class to the genetic etiology of each cardiomyopathy. The etiological fraction (EF) (x-axis) is an estimate of the proportion of affected carriers where the variant caused the disease; it is a measure of the interpretability of variants of this class (see **Supplementary Table S4a,b** online for full details). This measure is an average of all variants of a given class; some of which will be pathogenic but others will be benign.

At the gene level, ORs for nontruncating variants are typically modest; in the absence of prior clinical experience or functional data, interpretation is often uncertain. This may be improved by considering protein topology because pathogenic variants often cluster in specific regions in cases.^31,32^ We evaluated the distribution of rare missense alleles in *MYH7*, which encodes a protein with well-characterized functional and structural domains to determine whether systematic analysis of variant distribution refines interpretation. Nonrandom mutation cluster analysis^13^ revealed a significant cluster (*P* < 3 × 10^−15^; false discovery rate q < 5 × 10^−13^) between residues 181 and 937 in HCM cases, whereas in ExAC variants were depleted in this region and instead clustered between residues 1,271 and 1,903 (*P* < 3 × 10^−8^; false discovery rate q < 4 × 10^−5^) (**Figure 3a**). These data more precisely define the boundaries of mutation-enriched and depleted zones that can be used to generate more discriminating EFs; for example, for rare variants in HCM patients, EFs range from 0.97 in the HCM cluster to 0.67 in the control cluster (**Figure 3a**,**b**).

### Application of findings

To facilitate the application of these findings for research and clinical use, we provide an overview of the genetic landscape of cardiomyopathy as represented by patient referrals received by UK and US clinical testing laboratories. This shows the relative importance of cardiomyopathy genes within these patient populations (measured as a “case excess”) and their interpretability (expressed as the etiological fraction) (**Figure 3b**,**c**). Furthermore, we have created a Web resource, Atlas of Cardiac Genetic Variation (http://cardiodb.org/ACGV), to aid those assessing the relevance of specific genes and classes of variants to cardiomyopathies.

### Reassessing extended gene panel cardiomyopathy research studies

Several research studies^14,15,17,30^ using extended gene panels have reported genetic overlap between diverse cardiac diseases that appear at odds with known disease mechanisms. We surmise that many such studies have not adequately accounted for background genetic variation, have relied on variant data from incompletely annotated disease-centered databases, and have not used segregation. Here, using ExAC data, we present a reanalysis of two representative research studies.^14,15,16,17^

**Figure 4:**
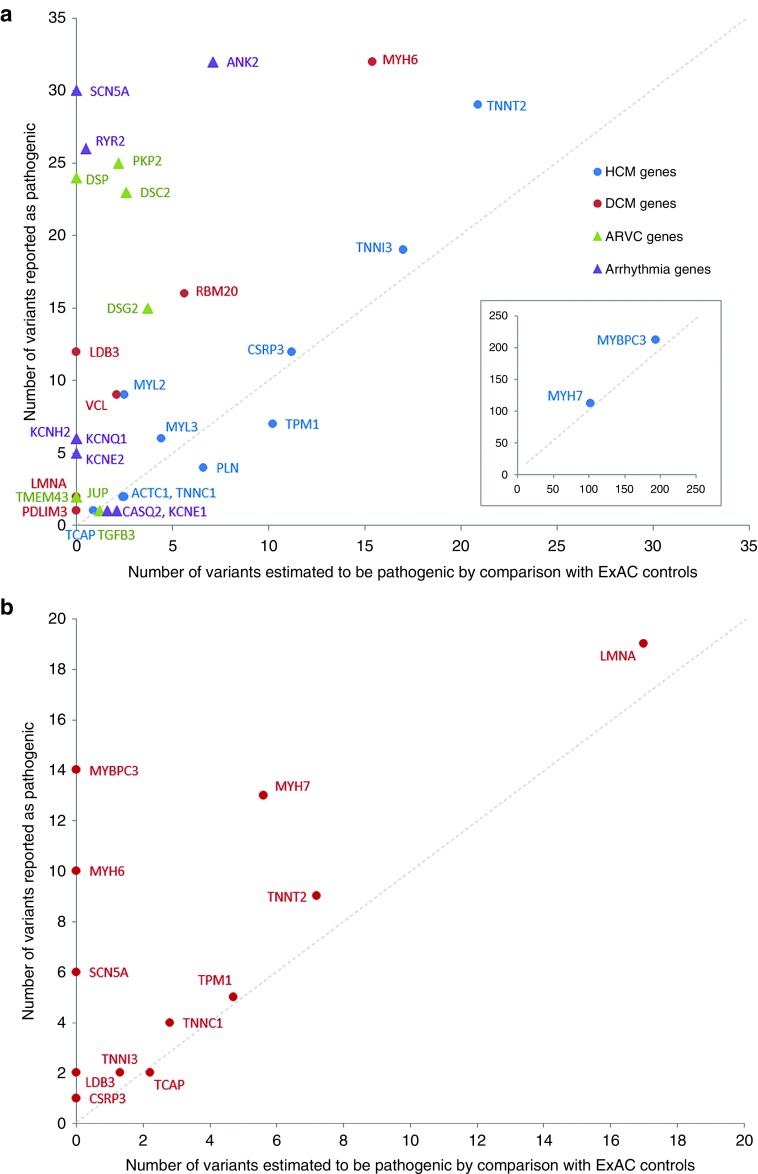
**Comparison of the number of variants reported as putatively pathogenic in research studies.** Hypertrophic cardiomyopathy (HCM) (**a**) and dilated cardiomyopathy (DCM) (**b**) in research studies (using generic analysis criteria such as variant class, missense-effect predictions, and variant population frequency in the Exome Sequencing Project) with those predicted as pathogenic by the excess of variation in cases over Exome Aggregation Consortium (ExAC) in each gene. For the HCM study^14^ (**a**), genes are colored according to the cardiac disease for which they are primarily associated, as defined by Lopes et al.^14^ Although there is good concordance between the research findings and the ExAC predictions for established HCM genes, for genes primarily associated with DCM, ARVC, and arrhythmias, the variation in cases is similar to that in ExAC. In the DCM study^15,16,17^ (**b**), variation burden in MYBPC3, SCN5A, and MYH6 is similar between the published research cases and ExAC, suggesting that most variants in these genes are unlikely to cause DCM.

In the research HCM cases, the excess variation in the known HCM genes (e.g., *MYBPC3*, *MYH7*) is substantial. By contrast, the measured variation in DCM, ARVC, and ion channel genes in the HCM patients—although also substantial—is similar to that seen in ExAC with little, if any, excess burden (**Figure 4a** and **Supplementary Table S6** online). This suggests that the majority of these variants, although individually rare, are benign bystanders, and that any overlap between the disorders has been overestimated.

In the DCM research studies, some genes proposed on the basis of these and other recent studies as among the most common causes of DCM (e.g., *MYBPC3*, *MYH6*, and *SCN5A*^15,17,30^) in fact showed no excess variation (**Figure 4b** and **Supplementary Table S7** online).

### Reassessment of variants previously reported as pathogenic

We examined the ExAC frequency of variants previously reported to cause HCM, DCM, or ARVC, as catalogued by HGMD (**Supplementary Tables S8–S10** online). A substantial number of purported disease-causing variants for HCM (25.2%; 322/1,280), DCM (29.2%; 222/759), and ARVC (34.6%, 167/483) were observed in ExAC. Although presence in ExAC does not preclude pathogenicity, a significant number are present at a frequency incompatible with causation of penetrant cardiomyopathy (6.5% of HCM, 11.9% of DCM, and 13.5% of ARVC variants are present at MAF >1 × 10^−4^; **Supplementary Tables S11 and S12** online). Of HGMD variants that could not be excluded as disease-causing using the Exome Sequencing Project (the largest control data set prior to ExAC) due to an allele count of one, 75% can now be discounted by ExAC refinement. In total, 11.7, 19.6, and 20.1% of individuals in ExAC have reported HCM, DCM, and ARVC variants, respectively; this is far in excess of disease prevalence. Hence, variant prioritization based on HGMD status alone is not advised for cardiomyopathy genes, a fact that is increasingly apparent with larger control data sets.

## Discussion

We present an analysis of data from 7,855 individuals referred for clinical diagnostic testing for inherited cardiomyopathies, along with 60,706 ExAC reference samples. These data exemplify the many challenges of variant interpretation in genetically heterogeneous disorders. We propose that in the absence of a large matched case–control series, the approaches described here, using data from large patient cohorts and broader reference data sets such as ExAC, may be applied to a range of multigenic, multiallelic diseases.

We show that the pathogenicity of disease genes originally identified through family linkage is resoundingly validated, for example, the majority of sarcomere genes in HCM. However, genes implicated in cardiomyopathy through candidate gene studies, including genes on panel tests in routine clinical use, are often not convincingly associated with disease. For example, *MYBPC3*, *MYH6*, and *SCN5A* have all been reported to be major contributors to DCM^15,17,30^ but show little or no excess burden despite adequate numbers and power; instead, we see that these are in fact genes that have the highest background variation.

We also show that it is crucial not only to distinguish variant classes but also to assess these in light of known disease mechanisms for each gene and disorder. For example, cardiomyopathy-causing variant in most myofilament proteins incorporate into the sarcomere and act as dominant negatives (HCM mutations are activating, whereas DCM mutations decrease myofibrillar function).^33^ Hence, protein-truncating variants that do not incorporate would not be expected to cause these conditions, and this is borne out in our data. By contrast, *MYBPC3* truncation alleles cause HCM through haploinsufficiency, making it unlikely that they could also cause DCM, which we confirm with our findings.

We summarize our analyses of cardiomyopathy genes in two measures, capturing the contribution of each gene to a disease (case excess) and our ability to interpret variation in each gene (etiological fraction (EF)). EF can be interpreted as the proportion of affected carriers in which the variant caused the disease (i.e., the proportion of true positives). EF is based on pooled rare variant frequency data, so it summarizes the average risk across many variants in a gene (some of which will be pathogenic but others will be benign) and will be particularly useful for selecting panels of genes that are informative for discrete phenotypes. Of critical importance, the probability that a novel variant is pathogenic depends on the clinical status of the individual carrying the variant and will be considerably lower in individuals with a remote/unrelated clinical diagnosis. This will be even more problematic with incidental findings during exome or genome sequencing with major implications for the recommendations to return apparently actionable findings.^8^

Although detailed phenotyping of the clinical cases was not available, we are confident that the clinical diagnoses are robust because the current clinical practice is to test only individuals with a confirmed diagnosis.^19,20^ The proportion of cases with inherited cardiomyopathy is unknown because evidence of familial disease is not a requirement for testing. The clinical sensitivity (proportion of patients with a pathogenic variant) in our case series was lower than that of previous surveys, which may reflect more restricted testing of stringently selected cases, typically from multiply affected families with severe disease.^34,35^ However, the cohorts studied are representative of those encountered by clinical diagnostic laboratories, rather than a highly selected subset.

Despite high levels of confidence in interpreting many well-characterized variants (which may give ORs in the hundreds), diagnostic laboratories are understandably cautious when interpreting a variant that has not been seen before. Our analyses demonstrate that for many genes even variants currently reported with variants of uncertain significance show a several-fold excess over the background in ExAC (**Figure 1**). More refined interpretation of variants in validated genes, for example, leveraging domain information, could increase the diagnostic yield of genetic testing and is likely to lead to much more substantial gains than the expansion of gene panels.

In contrast to the conservative strategy of clinical laboratories, research studies often report large “yields.” Some may not adequately control for the background rate of rare variation or may include genes for other conditions; as a result, genes nominated as important contributors to disease have little if any excess variation in cases. Testing of broad gene panels and overly inclusive interpretation of variants may lead to erroneous conclusions about pleiotropic effects of genetic variation^14,30^ and overestimates of double/compound mutations^36^ and the population prevalence of the disease.^37^

Despite the absence of demonstrable excess of rare variation in a gene, specific variants identified in family studies may still be disease-causing. However, if such variants are a small minority of rare variants in cases, then testing will yield more false positives than true positives. For some of the genes that show no excess (**Figure 1**), the original reports did not include any variant with robust evidence of segregation (i.e., LOD >3), and the possibility exists that the reported disease association is entirely spurious. An argument is often made that variants could be contributing as modifiers.^38,39^ This remains possible; however, in the absence of any significant overrepresentation in cases, the more parsimonious interpretation is that they are phenotypically silent. We have not tested more common variants (MAF >1 × 10^−4^) that could be mechanistically informative but are likely to have smaller effects,^40^ and we have not evaluated individual-level data to assess the impact of coinheritance of variants, which are limitations of the analyses.

A further limitation of this study is that the case and ExAC data were not generated using a single sequencing method; none of the methods used was expected to have 100% sensitivity for variant detection. Although these technical limitations could have marginal effects on estimates of rare variant frequency and OR/EF values, we do not expect them to alter the key conclusions of this study. Because ethnicity data were not available for the Oxford Medical Genetics Laboratory or LMM HCM patients, we were unable to confirm the extent to which the cohorts used in this study were matched by race. However, by studying the aggregate burden of multiple very rare variants, we expect that any confounding effects by individual population-specific variants in cases or ExAC will be limited. Supporting this assumption, an analysis of the LMM DCM cohort comparing findings from all populations with the Caucasian-only subsets revealed that the conclusions are robust (**Supplementary Table S13** online).

In conclusion, we have demonstrated that new opportunities for large-scale comparison of rare variation in Mendelian disease genes between patient cohorts and the wider population can identify the genes, regions of genes, and/or classes of variants that can be reliably interpreted in a clinical setting. For validated disease genes, there is clear potential to increase the yield of correctly interpreted, actionable variants. At the same time, problems must be avoided by recognizing that many implicated genes, as well as a significant proportion of variants, may not be robust. As clinical genetic testing moves to ever-larger gene panels and whole-exome and genome sequencing, an understanding of gene and variant pathogenicity will be increasingly important to deliver reliable interpretation.

## Data Availability

Web resource Atlas of Cardiac Genetic Variation (http://cardiodb.org/ACGV)

## Disclosure

Professor Stuart Cook occasionally consults for Illumina Inc. The other authors declare no conflict of interest.

## Figures and Tables

**Table 1:**
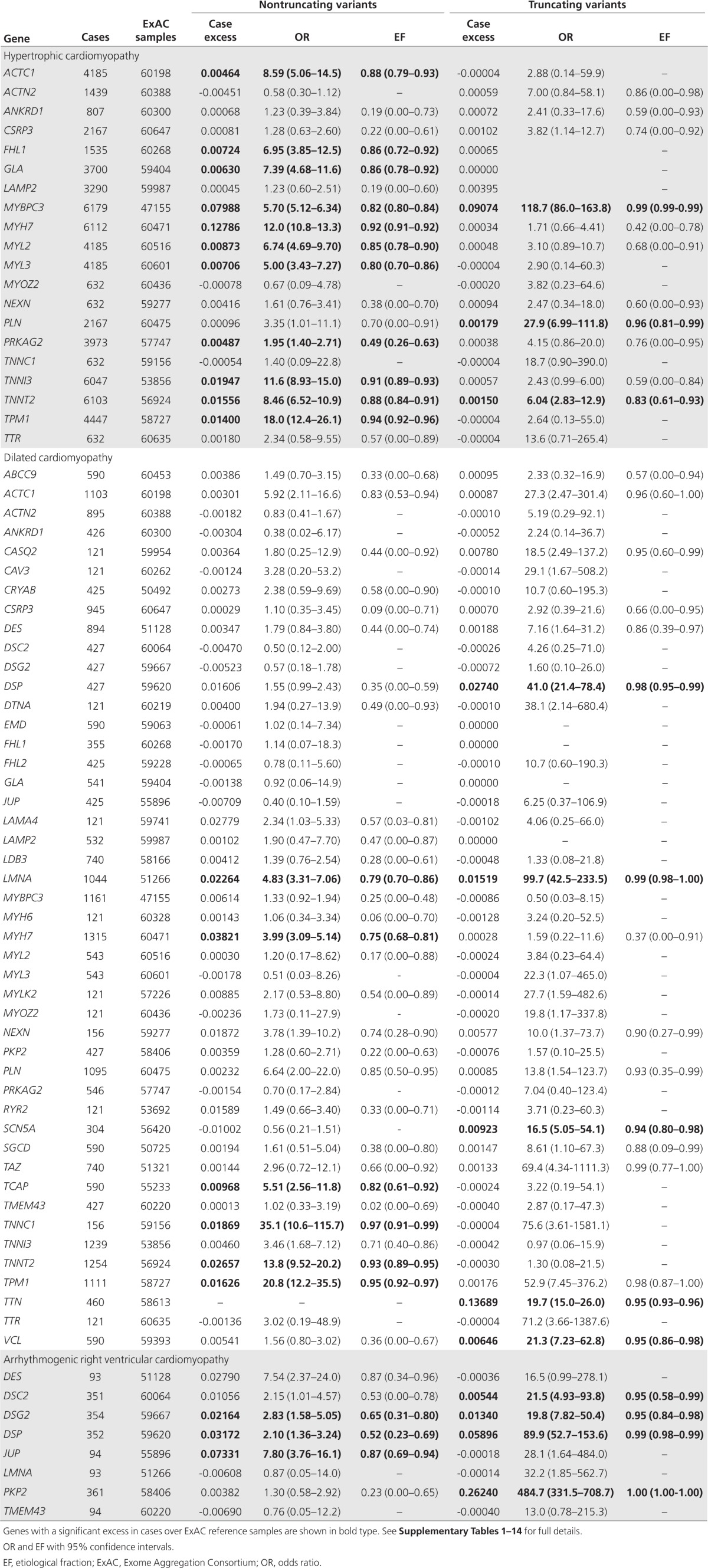
Summary of the study results, including the number of cardiomyopathy cases and ExAC reference samples analyzed, case excess, OR, and EF for nontruncating and truncating variants
