# Supplementary material for: Reassessment of Mendelian gene pathogenicity using 7,855 cardiomyopathy cases and 60,706 reference samples
Source: Genet Med. 2016 Aug 17;19(2):192–203. doi: 10.1038/gim.2016.90 (PMC5116235; doi:10.1038/gim.2016.90)
Supplement: Supplementary Information [file gim201690x1.zip › Supplementary Note 5.docx]

**Supplementary Note 5: Exome Aggregation Consortium**

Monkol Lek^1,2,3,4^, Konrad J Karczewski^1,2^, Eric V Minikel^1,2,5^, Kaitlin E Samocha^1,2,6,5^, Eric Banks^2^, Timothy Fennell^2^, Anne H O'Donnell-Luria^1,2,7^, James S Ware^2,8,9,10,11^, Andrew J Hill^1,2,12^, Beryl B Cummings^1,2,5^, Taru Tukiainen^1,2^, Daniel P Birnbaum^2^, Jack A Kosmicki^1,2,6,13^, Laramie E Duncan^1,2,6^, Karol Estrada^1,2^, Fengmei Zhao^1,2^, James Zou^2^, Emma Pierce-Hoffman^1,2^, Joanne Berghout^14,15^, David N Cooper^16^, Nicole Deflaux^17^, Mark DePristo^18^, Ron Do^19,20,21,22^, Jason Flannick^2,23^, Menachem Fromer^1,6,24,19,20^, Laura Gauthier^18^, Jackie Goldstein^1,2,6^, Namrata Gupta^2^, Daniel Howrigan^1,2,6^, Adam Kiezun^18^, Mitja I Kurki^2,25^, Ami Levy Moonshine^18^, Pradeep Natarajan^2,26,27,28^, Lorena Orozco^29^, Gina M Peloso^2,27,28^, Ryan Poplin^18^, Manuel A Rivas^2^, Valentin Ruano-Rubio^18^, Samuel A Rose^6^, Douglas M Ruderfer^24,19,20^, Khalid Shakir^18^, Peter D Stenson^16^, Christine Stevens^2^, Brett P Thomas^1,2^, Grace Tiao^18^, Maria T Tusie-Luna^30^, Ben Weisburd^2^, Hong-Hee Won^31^, Dongmei Yu^6,27,25,32^, David M Altshuler^2,33^, Diego Ardissino^34^, Michael Boehnke^35^, John Danesh^36^, Stacey Donnelly^2^, Roberto Elosua^37^, Jose C Florez^2,26,27^, Stacey B Gabriel^2^, Gad Getz^18,26,38^, Stephen J Glatt^39,40,41^, Christina M Hultman^42^, Sekar Kathiresan^2,26,27,28^, Markku Laakso^43^, Steven McCarroll^6,8^, Mark I McCarthy^44,45,46^, Dermot McGovern^47^, Ruth McPherson^48^, Benjamin M Neale^1,2,6^, Aarno Palotie^1,2,5,49^, Shaun M Purcell^24,19,20^, Danish Saleheen^50,51,52^, Jeremiah M Scharf^2,6,27,25,32^, Pamela Sklar^24,19,20,53,54^, Patrick F Sullivan^55,56^, Jaakko Tuomilehto^57^, Ming T Tsuang^58^, Hugh C Watkins^59,44^, James G Wilson^60^, Mark J Daly^1,2,6^, Daniel G MacArthur^1,2^

^1^Analytic and Translational Genetics Unit, Massachusetts General Hospital, Boston, MA, USA

^2^Program in Medical and Population Genetics, Broad Institute of MIT and Harvard, Cambridge, MA, USA

^3^School of Paediatrics and Child Health, University of Sydney, Sydney, NSW, Australia

^4^Institute for Neuroscience and Muscle Research, Childrens Hospital at Westmead, Sydney, NSW, Australia

^5^Program in Biological and Biomedical Sciences, Harvard Medical School, Boston, MA, USA

^6^Stanley Center for Psychiatric Research, Broad Institute of MIT and Harvard, Cambridge, MA, USA

^7^Division of Genetics and Genomics, Boston Children's Hospital, Boston, MA, USA

^8^Department of Genetics, Harvard Medical School, Boston, MA, USA

^9^National Heart and Lung Institute, Imperial College London, London, UK

^10^NIHR Royal Brompton Cardiovascular Biomedical Research Unit, Royal Brompton Hospital, London, UK

^11^MRC Clinical Sciences Centre, Imperial College London, London, UK

^12^Genome Sciences, University of Washington, Seattle, WA, USA

^13^Program in Bioinformatics and Integrative Genomics, Harvard Medical School, Boston, MA, USA

^14^Mouse Genome Informatics, Jackson Laboratory, Bar Harbor, ME, USA

^15^Center for Biomedical Informatics and Biostatistics, University of Arizona, Tucson, AZ, USA

^16^Institute of Medical Genetics, Cardiff University, Cardiff, UK

^17^Google Inc, Mountain View, CA, USA

^18^Broad Institute of MIT and Harvard, Cambridge, MA, USA

^19^Department of Genetics and Genomic Sciences, Icahn School of Medicine at Mount Sinai, New York, NY, USA

^20^Institute for Genomics and Multiscale Biology, Icahn School of Medicine at Mount Sinai, New York, NY, USA

^21^The Charles Bronfman Institute for Personalized Medicine, Icahn School of Medicine at Mount Sinai, New York, NY, USA

^22^The Center for Statistical Genetics, Icahn School of Medicine at Mount Sinai, New York, NY, USA

^23^Department of Molecular Biology, Massachusetts General Hospital, Boston, MA, USA

^24^Department of Psychiatry, Icahn School of Medicine at Mount Sinai, New York, NY, USA

^25^Psychiatric and Neurodevelopmental Genetics Unit, Massachusetts General Hospital, Boston, MA, USA

^26^Harvard Medical School, Boston, MA, USA

^27^Center for Human Genetic Research, Massachusetts General Hospital, Boston, MA, USA

^28^Cardiovascular Research Center, Massachusetts General Hospital, Boston, MA, USA

^29^Immunogenomics and Metabolic Disease Laboratory, Instituto Nacional de Medicina Gen—mica, Mexico City, Mexico

^30^Molecular Biology and Genomic Medicine Unit, Instituto Nacional de Ciencias M_dicas y Nutrici—n, Mexico City, Mexico

^31^Samsung Advanced Institute for Health Sciences and Technology (SAIHST), Sungkyunkwan University,Samsung Medical Center, Seoul, Republic of Korea

^32^Department of Neurology, Massachusetts General Hospital, Boston, MA, USA

^33^Vertex Pharmaceuticals, Boston, MA, USA

^34^Department of Cardiology, University Hospital, Parma, Italy

^35^Department of Biostatistics and Center for Statistical Genetics, University of Michigan, Ann Arbor, MI, USA

^36^Department of Public Health and Primary Care, Strangeways Research Laboratory, Cambridge, UK

^37^Cardiovascular Epidemiology and Genetics, Hospital del Mar Medical Research Institute, Barcelona, Spain

^38^Department of Pathology and Cancer Center, Massachusetts General Hospital, Boston, MA, USA

^39^Psychiatric Genetic Epidemiology & Neurobiology Laboratory, State University of New York,Upstate Medical University, Syracuse, NY, USA

^40^Department of Psychiatry and Behavioral Sciences, State University of New York,Upstate Medical University, Syracuse, NY, USA

^41^Department of Neuroscience and Physiology, State University of New York,Upstate Medical University, Syracuse, NY, USA

^42^Department of Medical Epidemiology and Biostatistics, Karolinska Institute, Stockholm, Sweden

^43^Department of Medicine, University of Eastern Finland and Kuopio University Hospital, Kuopio, Finland

^44^Wellcome Trust Centre for Human Genetics, University of Oxford, Oxford, UK

^45^Oxford Centre for Diabetes,Endocrinology and Metabolism, University of Oxford, Oxford, UK

^46^Oxford NIHR Biomedical Research Centre, Oxford University Hospitals Foundation Trust, Oxford, UK

^47^Inflammatory Bowel Disease and Immunobiology Research Institute, Cedars-Sinai Medical Center, Los Angeles, CA, USA

^48^Atherogenomics Laboratory, University of Ottawa Heart Institute, Ottawa, ON, Canada

^49^Institute for Molecular Medicine Finland (FIMM), University of Helsinki, Helsinki, Finland

^50^Department of Biostatistics and Epidemiology, Perelman School of Medicine at the University of Pennsylvania, Philadelphia, PA, USA

^51^Department of Medicine, Perelman School of Medicine at the University of Pennsylvania, Philadelphia, PA, USA

^52^Center for Non-Communicable Diseases, Karachi, , Pakistan

^53^Friedman Brain Institute, Icahn School of Medicine at Mount Sinai, New York, NY, USA

^54^Department of Neuroscience, Icahn School of Medicine at Mount Sinai, New York, NY, USA

^55^Department of Genetics, University of North Carolina, Chapel Hill, NC, USA

^56^Department of Medical Epidemiology and Biostatistics, Karolinska Institutet, Stockholm, Sweden

^57^Department of Public Health, University of Helsinki, Helsinki, Finland

^58^Department of Psychiatry, University of California, San Diego, CA, USA

^59^Radcliffe Department of Medicine, University of Oxford, Oxford, UK

^60^Department of Physiology and Biophysics, University of Mississippi Medical Center, Jackson, MS, USA
