# Supplementary material for: Reassessment of Mendelian gene pathogenicity using 7,855 cardiomyopathy cases and 60,706 reference samples
Source: Genet Med. 2016 Aug 17;19(2):192–203. doi: 10.1038/gim.2016.90 (PMC5116235; doi:10.1038/gim.2016.90)
Supplement: Supplementary Information [file gim201690x1.zip › Supplementary_Information_resubmit.docx]

**Supplementary Materials**

Supplementary Note 1: Details of the clinical and population reference cohorts

Supplementary Note 2: Selecting an allele frequency threshold to define potentially pathogenic and penetrant variants in Mendelian conditions

Supplementary Note 3: Etiological fraction (EF)

Supplementary Note 4: Assessment of population-specific effects

Supplementary Fig. S1. Comparison of the excess of rare variants in LMM DCM cases over controls between all population analysis and Caucasians only.

Table S1A. Variants identified in HCM, DCM and ARVC patients tested at Oxford Medical Genetics Laboratories (OMGL).

Table S1B. Variants identified in HCM and DCM patients tested at Partners Healthcare Laboratory of Molecular Medicine (LMM)

Table S2. Genes and transcripts analysed in this study and the number of patients sequenced in each disease cohort.

Table S3A. Comparison of the frequency of pathogenic variants in tested genes between OMGL and LMM clinical laboratories for HCM cohorts

Table S3B. Comparison of the frequency of pathogenic variants in tested genes between OMGL and LMM clinical laboratories for DCM cohorts

Table S4A. Comparison of the frequency of rare variation (ExAC MAF < 0.0001) in clinical HCM cases compared to ExAC controls.

Table S4B. Comparison of the frequency of rare variation (ExAC MAF < 0.0001) in clinical DCM cases compared to ExAC controls.

Table S4C. Comparison of the frequency of rare variation (ExAC MAF < 0.0001) in clinical ARVC cases compared to ExAC controls.

Table S5A. Odds ratios and Fisher's Exact test results testing for significance of the excess of rare variation in HCM cases versus ExAC controls.

Table S5B. Odds ratios and Fisher's Exact test results testing for significance of the excess of rare variation in DCM cases versus ExAC controls.

Table S5C. Odds ratios and Fisher's Exact test results testing for significance of the excess of rare variation in ARVC cases versus ExAC controls.

Table S6. Frequency of rare variants in HCM research cohort with comparison between the number of variants reported as pathogenic by Lopes et al with the number predicted by the case excess observed versus ExAC controls.

Table S7. Frequency of rare variants in DCM research cohort with comparison between the number of variants reported as pathogenic by Hershberger et al with the number predicted by the case excess observed versus ExAC controls.

Table S8. Numbers of variants in the HGMD database (professional version 2015.1) associated with HCM by gene with number of these variants present in ExAC (at any frequency and greater than 0.0001), the Exome Sequencing Project (ESP) or 1000 Genomes (1KG) and the total number of ExAC alleles and individuals with a HCM-associated variant.

Table S9. Numbers of variants in the HGMD database (professional version 2015.1) associated with DCM by gene with number of these variants present in ExAC (at any frequency and greater than 0.0001), the Exome Sequencing Project (ESP) or 1000 Genomes (1KG) and the total number of ExAC alleles and individuals with a DCM-associated variant.

Table S10. Numbers of variants in the HGMD database (professional version 2015.1) associated with ARVC by gene with number of these variants present in ExAC (at any frequency and greater than 0.0001), the Exome Sequencing Project (ESP) or 1000 Genomes (1KG) and the total number of ExAC alleles and individuals with an ARVC-associated variant.

Table S11. Summary of the numbers of variants in the HGMD database (professional version 2015.1) associated with HCM, DCM and ARVC which are present in ExAC (at any frequency and greater than 0.0001), the Exome Sequencing Project (ESP) or 1000 Genomes (1KG) and the total number of ExAC alleles and individuals with a ARVC-associated variant.

Table S12. List of variants linked to cardiomyopathies (HCM, DCM, ARVC) in the HGMD database (professional version 2015.1) that have ExAC frequencies > 0.0001.

Table S13. Comparison of case-control analysis for LMM DCM cases with (1) Caucasian DCM patients versus ExAC Non-Finnish European subset and (2) All DCM patients versus total ExAC dataset.

Table S14. Power calculations.

# Supplementary Note 1

*Details of clinical and population reference cohorts*

| **Dataset** | **Cohort Type** | **Reference** | **Sample size** | **No. genes tested** | **Methods used to generate sequence data** |
| --- | --- | --- | --- | --- | --- |
| OMGL HCM | Clinical | this paper | 807 - 3267 | 16 | High-resolution DNA melting (WAVE dHPLC, LightScanner^®^)  Sanger sequencing  Targeted NGS |
| LMM HCM | Clinical | ^1^ | 632 - 2912 | 18 | DNA microarray (Cardiochip)  Sanger sequencing.  Targeted NGS |
| Combined HCM | Clinical | - | 632 - 6179 | 20 | As detailed above |
| OMGL DCM | Clinical | this paper | 304 - 559 | 28 | High-resolution DNA melting (WAVE dHPLC, LightScanner^®^)  Sanger sequencing  Targeted NGS |
| LMM DCM | Clinical | ^2^ | 121 - 756 | 46 | DNA microarray (Cardiochip)  Sanger sequencing  Targeted NGS |
| Combined DCM | Clinical | - | 121 - 1315 | 48 | As detailed above |
| OMGL ARVC | Clinical | this paper | 93 - 361 | 8 | High-resolution DNA melting (WAVE dHPLC, LightScanner^®^)  Sanger sequencing  Targeted NGS |
| Research HCM | Research | ^3^ | 874 | 36 | Targeted NGS |
| Research DCM | Research | ^4–6^ | 312 - 324 | 12 | Sanger sequencing |
| ExAC | Database | ^7^ | Up to 60706 | all | Exome sequencing |

**Table 2:** Overview of the datasets analyzed in this study. Clinical data is from the Oxford Medical Genetics Laboratories (OMGL), UK and the Laboratory of Molecular Medicine (LMM), USA (combined clinical datasets for HCM and DCM are shaded in grey).

**Oxford Medical Genetics Laboratory (OMGL)**

The OMGL cohort comprises apparently unrelated index cases referred from Clinical Genetics centers across the UK, with initial clinical diagnosis of HCM, DCM or ARVC made by a consultant cardiologist. All samples received for diagnostic genetic testing of HCM, DCM or ARVC genes were eligible and analysis was undertaken in a routine clinical setting using clinical consent. Comprehensive data on patient ethnicity is not available for this cohort. Genotype data was obtained from 3267 individuals with HCM, 559 with DCM and 361 with ARVC. The current panel sizes include 16 genes for HCM, 28 genes for DCM and 8 genes for ARVC. Not all patients were analyzed for every gene (see Table S2). Variants reported in this cohort were classified according to national guidelines (http://www.acgs.uk.com) as highly likely to be pathogenic (Class 5), likely to be pathogenic (Class 4), or variant of unknown significance (VUS) (Class 3).

**Laboratory of Molecular Medicine, Partners Healthcare (LMM)**

Data from LMM was downloaded from the supplemental files of published HCM^1^ (18 genes sequenced in 632 - 2912 patients) and DCM^2^ (46 genes sequenced in 121 - 756 patients) cohorts. The LMM HCM cohort comprised unrelated probands referred for HCM clinical genetic testing. Any individuals with an unclear clinical diagnosis of HCM, or with left ventricular hypertrophy due to an identified syndrome such as Fabry or Danon disease, or unaffected individuals with a family history of HCM were excluded. The LMM DCM cohort comprised individual probands referred for DCM clinical genetic testing. According to the published report, all patients had DCM or clinical features consistent with DCM based on the medical and family history information provided by ordering providers. Additionally, any cases with confirmed diagnoses of other cardiomyopathies, structural heart disease, congenital heart disease or syndromic or environmental causes were not included in the study. Variants are classified as pathogenic, likely pathogenic, VUS favor pathogenic or others (other VUS, likely benign) according to the LMM's clinical grade variant classification criteria^2^.

**Data from clinical cohorts**

OMGL and LMM use similar clinical guidelines and employ equivalent approaches for variant classification in line with published guidelines^8^, however different names are used for each class. In this manuscript the term Pathogenic (P), includes OMGL Class 5, and LMM Pathogenic; Likely pathogenic (LP), includes OMGL Class 4 and LMM Likely pathogenic; and Variant of Uncertain significance (VUS), includes OMGL Class 3 and the LMM VUS favor pathogenic and other VUS (see Fig. 1).

In these clinical cohorts, sequence data was generated using a range of mutation scanning and direct sequencing techniques of varying sensitivity (High-resolution DNA melting, WAVE dHPLC, LightScanner®, DNA microarray [Cardiochip], Sanger sequencing, targeted Next Generation Sequencing [NGS]). These targeted tests are designed to cover the coding regions and splice sites of the key genes of interest; the analytical sensitivity of these methods is estimated to be in the region of 98-100% (data from in house validation). As all putative pathogenic variants are confirmed by Sanger sequencing the rate of false positive variant calls will be negligible.

**Exome Aggregation Consortium (ExAC) cohort**

The ExAC dataset comprises aggregated sequencing data from a variety of large-scale exome sequencing projects, reprocessed through the same pipeline. Cohorts likely to be enriched for Mendelian disease were not included in this dataset and while cardiomyopathy has not been excluded in ExAC cases, the selection criteria for the component cohorts would not be expected to enrich for inherited cardiac conditions. VCF data was downloaded from the Exome Aggregation Consortium (ExAC), Cambridge, MA (http://exac.broadinstitute.org) [version 0.3, Jan 2015]. Quality control analyses suggest a sensitivity of 97-99.8% for single nucleotide variants (SNVs) and approximately 95% for insertions and deletions (indels)^7^. To minimize any bias resulting from the higher sensitivity strategies employed by diagnostic clinical laboratories, only genes with a high proportion of coding region covered to a median sequence depth of >30x and only high quality (PASS filter) variants were included in our analyses. In addition we adjusted the total number of ExAC samples per gene based on the mean coverage at the variant sites of interest.

The sample size was fixed by the availability of clinically sequenced and ExAC samples. Illustrative power calculations are reported in Supplementary Table S14.

# Supplementary Note 2

## Selecting an allele frequency threshold to define potentially pathogenic and penetrant variants in Mendelian conditions

How many times must a variant be observed in ExAC to consider it too common to cause a Mendelian cardiomyopathy?

Clearly, a penetrant Mendelian allele should not be present in an unselected population more frequently than the disease it causes. Moreover, for a genetically heterogeneous condition it must not be more frequent than the proportion of cases attributable to that gene, or indeed to any single variant. Given the substantial datasets presented here we can now estimate these proportions reasonably robustly, but must take care to provide an appropriate margin of error given that these estimates are derived from samples of fixed size and ethnicity.

HCM has an estimated prevalence of 1:500^9^. In this series the variant to which the largest proportion of cases is attributable is MYBPC3 c.1504C>T (p.Arg502Trp), found in 104/6179 HCM cases (1.7%, 95CI 1.4-2.0%). Caution must be applied when considering calculating confidence intervals for allele frequencies, especially for very rare alleles, as the underlying distribution of allele frequencies from which our sample is drawn is not fully known, and strongly left skewed (e.g. a singleton variant is much more likely to have a true frequency below the measured frequency than above it). Nonetheless, the binomial distribution is a fair approximation at this allele frequency range, and will generally be conservative when calculating upper confidence intervals for the frequency of rare alleles, though not at all robust for calculating lower confidence intervals.

Given that MYBPC3c.1504C>T is seen in 1.7% of cases (in the heterozygous state), and cases have a prevalence of 1:500, we expect a population allele frequency around 1.7 x10-5.

104/6179 x 1/500 x 1/2 (as each individual is diploid) = 1.7x10-5

We can cross-reference this against ExAC. This variant is observed 3 times in ExAC (in 60557 individuals genotyped at this site), and twice in 33329 Europeans (non-Finnish). This gives an observed ExAC global MAF = 2.5x10-5 (6.0x10-5 in Europeans), and an upper bound for the true population frequency of this allele would be estimated as 5.3 x10-5 based on a binomial distribution around the ExAC global MAF, or 7.2x10-5 based on the European subset.

# upper limit based on observation in global ExAC
binom::binom.confint(3,2*60557,methods="asymptotic",confint=0.95)$upper

## [1] 5.279914e-05

# upper limit based on observation in ExAC Europeans
binom::binom.confint(2,2*33329,methods="asymptotic",confint=0.95)$upper

## [1] 7.15858e-05

So we consider that a variant with a true population allele frequency > 7 x 10-5 as too common to cause HCM. We may want to adjust this threshold to allow for reduced penetrance. We could simply divide this maximum frequency by the penetrance of the condition (e.g. a variant with a penetrance of 0.5 could be present at double this frequency).

In fact we have made a series of conservative assumptions (and more are to come), so have not made an additional correction for reduced penetrance at this point.

Finally, we ask how many times such a variant might be a variant with true population allele frequency 7x10-5 be observed in a random population sample. This can be modeled using a poisson distribution: for a 5% error rate we take the 95th centile of a poisson distribution with λ = expected allele count given by 2 x sample size x population allele frequency:

maxAF=7E-5
myCI=0.95
nSamples=60706
alleleNumber=2*nSamples
maxAC = qpois(myCI, alleleNumber*maxAF)

maxAC

## [1] 14

This allows us to adjust our threshold for variants that are not sequenced in the entire cohort (indicated by the “AN” field in ExAC), or by focusing on one ethnic subgroup. For example, if the variant was found only in East Asians, we could derive an ExAC AC cut-off as follows:

nSamples=4327 # (ExAC EAS)
qpois(myCI,2*nSamples*maxAF)

## [1] 2

**Limitations**

In the populations studied, a measured AF greater than this threshold is incompatible with penetrant Mendelian cardiomyopathy. However, deleterious founder variants may be present in other populations in whom the genetic architecture of CM is undefined. As described here, we have not accounted for reduced penetrance and thresholds would need to be adjusted for models in which this was included. The calculations are also dependent on the stated prevalence of HCM (1 in 500), although we expect this to be a robust estimate of the disease frequency.

The maximum allele frequency that is compatible with pathogenicity will be different for each condition according to its prevalence and the known genetic architecture of the disease.

Examples here are generated using the R statistical environment.

# Supplementary Note 3

*Etiological fraction (EF)*

*Attributable risk percent among exposed* (ARP), provides an estimate of the proportion of the risk in an exposed population that can be attributed to the exposure. This epidemiological measure is meaningful where there is strong evidence of a biologically plausible causal relationship between exposure and disease. In our context, ARP corresponds to the proportion of the risk of cardiomyopathy in mutation carriers that can be attributable to the mutation. The measure was popularized by Cole & MacMahon^10^, and defined as follows:

$$ARP\left( \% \right)= \frac{R_{e}-R_{0}}{R_{e}}\times100\%$$

where
$R_{e}$ = risk in the exposed (i.e. carriers of rare variant in gene of interest)
$R_{o}$ = risk in the unexposed (i.e. individuals with no rare variant in gene of interest)

The equation can be conveniently rewritten as:

$$ARP(\%)=\frac{RR-1}{RR}\times100\%$$

where
$RR$ = relative risk (ratio of risk among exposed to risk among unexposed) (Robins & Greenland ^11^)

For cross-sectional (i.e. case-control) data, odds ratios (OR) provide accurate estimates of the underlying relative risk ^12^, leading to:

$$ARP(\%)=\frac{OR-1}{OR}\times100\%$$

Now ARP, when expressed as a decimal fraction, has been called the etiological fraction (EF) ^11^ i.e.

$$EF=\frac{OR-1}{OR}$$

and we refer to EF hence-with.

In the diagnostic context, where we are treating cardiomyopathy as a Mendelian disease, and are interested in interpreting whether an individual rare variant was likely causative, the EF can be interpreted in several ways**^a^**:

- the proportion of variant-carrying probands in which the variant was causal
- the proportion of variants, found in affected individuals, that were penetrant
- the probability that an individual rare variant, found in a proband, was responsible for the disease

EF therefore represents our confidence in interpreting variation as etiologically significant when found in an individual with disease.

**a**It is worth noting that in each case we cannot disentangle incomplete penetrance. Without additional information we cannot determine whether an EF of 50% indicates that half of the variants found in our case cohort are fully penetrant and disease-causing (and the other half have zero penetrance), or if all of the variants are pathogenic, but with reduced (50%) penetrance.

# Supplementary Note 4

*Assessment of population-specific effects*

As ethnicity data was not available for the OMGL cases or the LMM HCM cohort, the total case and control datasets were used in this study. ExAC is a mixed dataset but with a majority of samples of European descent (52% non-Finnish European) which should be well matched to the LMM cohorts (62% white/Caucasian) and the unselected UK referral population of the OMGL cohorts. To assess if population-specific variants had any confounding effects, we compared the results observed between the LMM DCM cohort (for which individual self-reported patient ethnicity data is available) and ExAC for the full datasets and for white DCM patients (456 samples) versus the ExAC non-Finnish European subset (33,370 samples). Discrepancies were defined as any gene which had a significant case excess (as defined above) in one of the analyses but not the other. The correlation (Pearson coefficient) between case excess frequencies observed in both analyses was also calculated for genes with more than 300 samples sequenced. There was full correlation between the genes that were significantly enriched in both comparisons (Table S13), with a Pearson correlation coefficient of 0.97 between the case excess values for genes in the two comparisons (Supplementary Fig S1), showing that population stratification effects are not a confounder in this study.

Supplementary Figure S1.


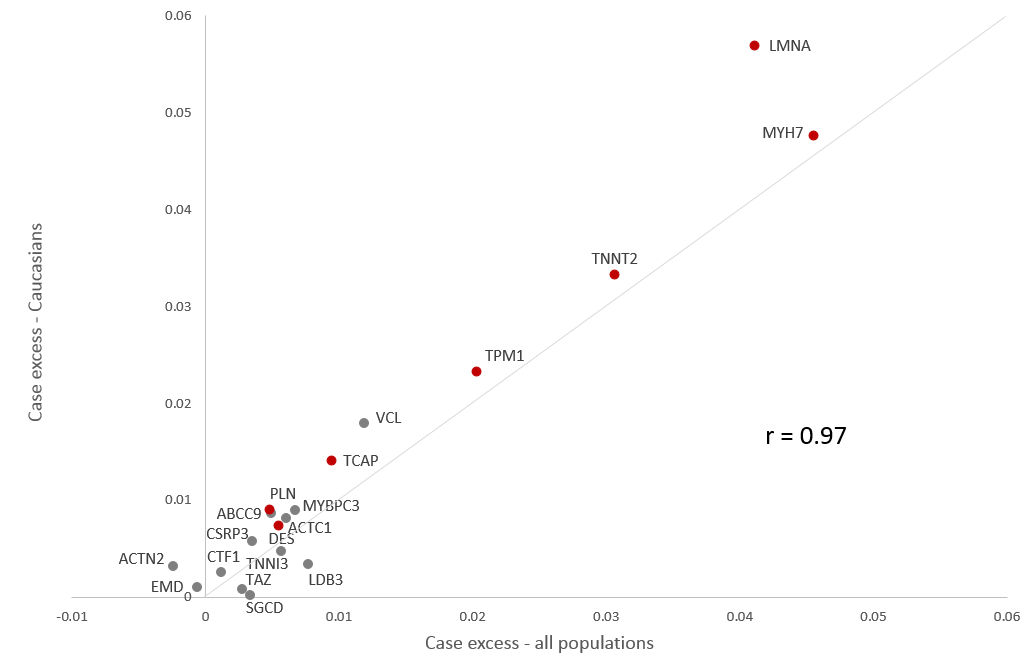


# Figure S1: Comparison of the excess of rare variants in LMM DCM cases over controls between all population analysis (all cases versus full ExAC dataset) and Caucasian only (Caucasian DCM cases versus non-Finnish European subset of ExAC). Genes with a significant excess in cases (Fisher’s exact test p<0.001 with Bonferroni correction for 48 tests) are shown in red. The Pearson correlation coefficient was calculated at 0.97. Only genes with >300 samples sequenced are shown.

**REFERENCES**

1. Alfares A a., Kelly M a., McDermott G, et al. Results of clinical genetic testing of 2,912 probands with hypertrophic cardiomyopathy: expanded panels offer limited additional sensitivity. *Genet Med*. 2015;(January). doi:10.1038/gim.2014.205.

2. Pugh TJ, Kelly M a, Gowrisankar S, et al. The landscape of genetic variation in dilated cardiomyopathy as surveyed by clinical DNA sequencing. *Genet Med*. 2014;16(8):601-608. doi:10.1038/gim.2013.204.

3. Lopes LR, Syrris P, Guttmann OP, et al. Novel genotype-phenotype associations demonstrated by high-throughput sequencing in patients with hypertrophic cardiomyopathy. *Heart*. 2014;101(4):294-301. doi:10.1136/heartjnl-2014-306387.

4. Hershberger RE, Norton N, Morales A, Li D, Siegfried JD, Gonzalez-Quintana J. Coding sequence rare variants identified in MYBPC3, MYH6, TPM1, TNNC1, and TNNI3 from 312 patients with familial or idiopathic dilated cardiomyopathy. *Circ Cardiovasc Genet*. 2010;3(2):155-161. doi:10.1161/CIRCGENETICS.109.912345.

5. Parks SB, Kushner JD, Nauman D, et al. Lamin A/C mutation analysis in a cohort of 324 unrelated patients with idiopathic or familial dilated cardiomyopathy. *Am Heart J*. 2008;156(1):161-169. doi:10.1016/j.ahj.2008.01.026.

6. Hershberger RE, Parks SB, Kushner JD, et al. Coding sequence mutations identified in MYH7, TNNT2, SCN5A, CSRP3, LBD3, and TCAP from 313 patients with familial or idiopathic dilated cardiomyopathy. *Clin Transl Sci*. 2008;1(1):21-26. doi:10.1111/j.1752-8062.2008.00017.x.

7. Exome Aggregation Consortium, Monkol Lek, Konrad Karczewski, Eric Minikel, Kaitlin Samocha, Eric Banks, Timothy Fennell, Anne O’Donnell-Luria, James Ware, Andrew Hill, Beryl Cummings, Taru Tukiainen, Daniel Birnbaum, Jack Kosmicki, Laramie Duncan, Karol E DM. Analysis of protein-coding genetic variation in 60,706 humans. *In-press*. http://biorxiv.org/content/early/2015/10/30/030338.

8. Richards S, Aziz N, Bale S, Bick D, Das S. ACMG Standards and Guidelines Standards and guidelines for the interpretation of sequence variants : a joint consensus recommendation of the American College of Medical Genetics and Genomics and the Association for Molecular Pathology. 2015;17(January):1-20. doi:10.1038/gim.2015.30.

9. Maron BJ, Gardin JM, Flack JM, Gidding SS, Kurosaki TT, Bild DE. Prevalence of hypertrophic cardiomyopathy in a general population of young adults. Echocardiographic analysis of 4111 subjects in the CARDIA Study. Coronary Artery Risk Development in (Young) Adults. *Circulation*. 1995;92(4):785-789. doi:10.1161/01.CIR.92.4.785.

10. Cole P, MacMahon B. Attributable risk percent in case-control studies. *Br J Prev Soc Med*. 1971;25(4):242-244. doi:10.1136/jech.25.4.242.

11. Robins JM, Greenland S. Estimability and estimation of excess and etiologic fractions. *Stat Med*. 1989;8(7):845-859.

12. Lipsitz SR. Categorical data analysis. Alan Agresti, Wiley, New York, 1990. no. of pages: xv + 558. Price: £48.50, 72.95. ISBN: 0-471-85301-1. *Stat Med*. 1992;11(13):1791-1792. doi:10.1002/sim.4780111313.
